# Deformities of the Uvula in the Oral Cavity- A Case Series

**Published:** 2012-10-30

**Authors:** S Achalli, S Bhat, S Ram Shetty, S G Babu, R Suvarna

**Affiliations:** 1 Department of Oral Medicine and Radiology A.B. Shetty Memorial Institute of Dental Sciences,NITTE University, Mangalore, India

**Keywords:** Bifid uvula, Cleft, Uvula

## Abstract

**Background:**

Disorders of the palatal uvula is one of the least highlighted areas of medical literature, inspite the fact that uvula is a key organ in functions like speech, deglutition and mastication. The aim of this paper is to present a series of cases of wide range of uvular deformities ranging from bifid uvula to absence of uvula.

## Introduction

Uvula is the part which hangs down to rest on the dorsum of the tongue from the free posterior margin of the soft palate.(1) The uvula helps to prevent the soft palate being forced into the nasopharynx or mouth when it is resisting pressure differences between these and the oral part of the pharynx as in coughing or sneezing.(1)

The term cleft uvula signifies partial or total bifurcation of the uvula.(2) The least severe form of cleft palate is the bifid uvula. Increasingly severe clefts always pose posterior involvement, the cleft advancing anteriorly in contradistinction to the direction of normal fusion.(3) A cleft uvula has a fishtail appearance, or the cleft may extend through the soft and hard regions of the palate.(4) The prevalence of cleft uvula is much higher than that of cleft palate with a frequency of 1 in every 80 white individuals.(5) Its occurrence has attracted increased attention during the recent years because of the assumption that cleft uvula is a microform of cleft palate.(2)

Glossopharyngeal nerve is the IX cranial nerve, which helps the palatal movement, as it innervates the stylopharyngeus muscles (having a role in elevation of the pharynx), the damage of which can result in a complication called glossopharyngeal nerve palsy.(6)

## Case Report

Case 1: A 28-year-old male patient referred to Department of Oral Medicine and Radiology with a complaint of decay in the upper left back tooth for one year. The patient presented a history of defective speech and lisping from childhood.

On general examination the patient was moderately built and nourished. There was no relevant medical and family history.

**Figure 1:**
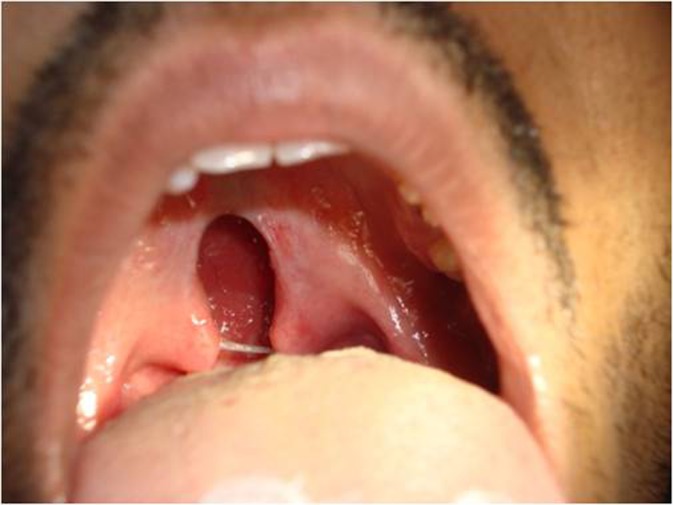
Cleft involving the uvula bifurcated from three-fourths to its total length-Type D according to Meskin et al classification 1964

On intraoral examination no abnormalities were detected in the palate. The uvula was inspected after flattening the tongue with the dental mirror. Cleft involving the uvula bifurcated from three-fourths to its total length was detected (Figure.1). A provisional diagnosis of isolated bifid uvula was made (Type D) according to Meskin et al classification 1964.

**Figure 2:**
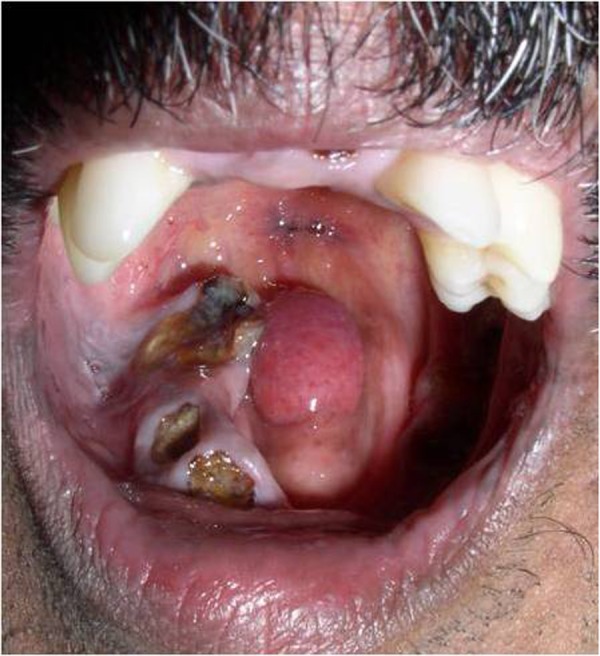
Palate showing absence of uvula

Case 2: A 42 year-old male referred to us with decayed teeth in the upper and lower jaws. The patient had difficulty in speech and swallowing from childhood due to an abnormally small tongue. He had no other relevant medical history. No other abnormalities were detected during general physical examination. Intraoral examination revealed presence of multiple root stumps in the maxillary and mandibular arches. The tongue was hypolastic, around 2 cms long antero-posteriorly and 1 cm wide. The tongue surface was devoid of papilla and movements were restricted in all directions. Lingual frenum was absent, and the floor of the mouth was pinkish in appearance and leathery. The mandibular arch was v-shaped. The palate was high arched with collapsed maxillary arch. Uvula and faucial pillars were ill-developed. The palate showed complete absence of uvula (Figure.2).

Case 3: A 24-year-old man referred to the outpatient department, with the chief complaint of difficulty in swallowing. He denied any history of trauma but gave us the history of tonsillectomy three years ago.

**Figure 3:**
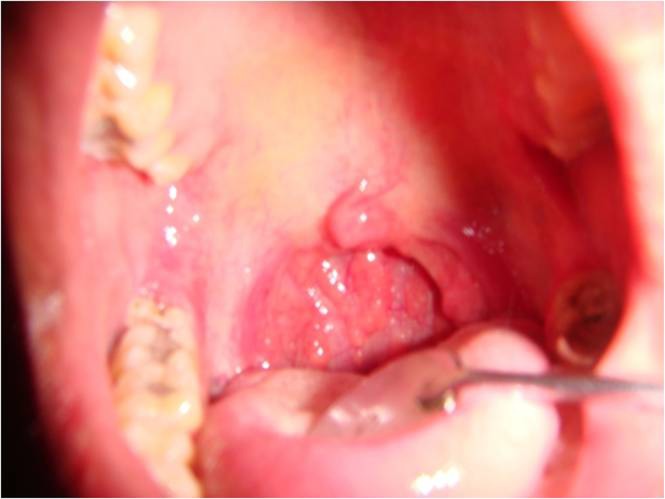
Uvular deviation seen to the right

Clinical examination showed incomplete elevation of the soft palate on the right side. There was absence of gag reflex and uvular deviation to the right was seen (Figure 3). No facial palsy, diplopia, nor any evidence of motor weakness or sensory deficit was present. A provisional diagnosis of uvular deviation to the right secondary to glossopharyngeal nerve palsy was made.

## Discussion

Cleft uvula has been reported to represent the least severe form of cleft palate.(3) Various studies have shown the prevalence of cleft uvula associated with cleft palate to ranging between 1.5 and 10% but isolated cleft uvula has rarely been reported. It is a symptom of several chromosomal syndromes (eg. Trisomy). The lesion may possibly be genetically inherited. Bifid uvula is more common in males than in females.(7) Formation of soft palate and uvula takes a slightly different course than that of the regions of the secondary palate which give rise to the hard palate.(8) The soft palate and uvula develop from two separate masses found at the most posterior portions of the secondary palatine shelves. Unlike the fusion mechanism which is in place along much of the length of the palatine shelves, the consolidation of these two separate masses is brought about by a selective proliferation of mesenchymal cells located deep in the valley between the masses. As that proliferation, called merging, continues the valley between the two distal shelf masses is obliterated, this results in the smoothening of the contour of the soft palate and uvula. Failure of the merging process in the soft palate and uvula development can result in complete or partial clefts of the soft palate and uvula.(9)

Classification of the types of cleft uvula according to Meskin et al (1964) based on morphology are: Type A: Normal uvula, Type B: Uvula bifurcated upto one-fourth of its total length, Type C: Uvula bifurcated from one-fourth to three-fourths of its length, and Type D: Uvula bifurcated from three-fourths to its total length.(10)

It is hypothesized that cleft uvula is inherited as an autosomal dominant trait with about 40% penetrance. It could be expected that of 50 families having a cleft uvula proband, 40% or 20 parents would demonstrate a similar defect. If cleft uvula is a true palatal cleft it should follow the hereditary pattern of severe cleft palate.(11)

Recent articles have shown the occurrence of bifid uvula in rare conditions like Loeys–Dietz syndrome and inflammatory linear verrucous epidermal nevus syndrome.(12,13)

Glossopharyngeal nerve or the IX cranial nerve helps the palatal movement and damage to this nerve can result in a complication called glossopharyngeal nerve palsy.(6) Direct nerve injury can be the most plausible explanation for this rare complication. Damage to the vagus nerve or the X cranial nerve can also cause impairment in pharyngeal movements like symmetric elevation of the soft palate and shortening of the uvula when the patient says ‘ah’.(6)

Bulbar palsy, occipital condyle fracture, traumatic dissection of internal maxillary artery and compression of the nerve by rheumatoid pannus can also be associated with isolated glossopharyngeal palsy.(14-16)

## Conclusion

It can be concluded that information regarding cleft uvula or absence of uvula can be of high importance in future studies of cleft palate etiology and in genetic counseling.
